# Automatic Detection of Liver Cancer Using Hybrid Pre-Trained Models

**DOI:** 10.3390/s22145429

**Published:** 2022-07-20

**Authors:** Esam Othman, Muhammad Mahmoud, Habib Dhahri, Hatem Abdulkader, Awais Mahmood, Mina Ibrahim

**Affiliations:** 1 Faculty of Applied Computer Science, King Saud University, Riyadh 11451, Saudi Arabia; eothman@ksu.edu.sa (E.O.); hdhahri@ksu.edu.sa (H.D.); mawais@ksu.edu.sa (A.M.); 2 Department of Information Systems, Madina Higher Institute of Management and Technology, Shabramant 12947, Egypt; m.hanafi@madinagroups.edu.eg; 3 Department of Information Systems, Faculty of Computers and Information, Menoufia University, Shebin El-kom 32511, Menoufia, Egypt; hatem.abdelkader@ci.menofia.edu.eg; 4 Department of Information Technology, Faculty of Computers and Information, Menoufia University, Shebin El-kom 32511, Menoufia, Egypt

**Keywords:** CNN, liver cancer, CT images, deep learning, pre-trained models

## Abstract

Liver cancer is a life-threatening illness and one of the fastest-growing cancer types in the world. Consequently, the early detection of liver cancer leads to lower mortality rates. This work aims to build a model that will help clinicians determine the type of tumor when it occurs within the liver region by analyzing images of tissue taken from a biopsy of this tumor. Working within this stage requires effort, time, and accumulated experience that must be possessed by a tissue expert to determine whether this tumor is malignant and needs treatment. Thus, a histology expert can make use of this model to obtain an initial diagnosis. This study aims to propose a deep learning model using convolutional neural networks (CNNs), which are able to transfer knowledge from pre-trained global models and decant this knowledge into a single model to help diagnose liver tumors from CT scans. Thus, we obtained a hybrid model capable of detecting CT images of a biopsy of a liver tumor. The best results that we obtained within this research reached an accuracy of 0.995, a precision value of 0.864, and a recall value of 0.979, which are higher than those obtained using other models. It is worth noting that this model was tested on a limited set of data and gave good detection results. This model can be used as an aid to support the decisions of specialists in this field and save their efforts. In addition, it saves the effort and time incurred by the treatment of this type of cancer by specialists, especially during periodic examination campaigns every year.

## 1. Introduction

Cancer is a general term that includes a wide range of diseases that can affect any part of the body. Cancer is considered a leading cause of death worldwide, claiming nearly 10 million lives in 2020, or approximately 1 in 6 deaths. One of the most common types of cancer is liver cancer. The American Cancer Society estimates that about 30,520 people (20,420 men and 10,100 women) will die of liver cancer [1] at the end of 2022. Liver cancer causes more deaths than other types of cancer, and the disease is usually diagnosed when it is in its advanced stages. Therefore, diagnosing this disease at an early stage leads to better treatment options. This paper proposes an efficient method for the early detection of liver cancer. Non-invasive procedures such as CT scans, MRI, and ultrasound are used to identify liver cancer. The CT scan is a type of X-ray that produces detailed images of your body. This scan can provide information on the size, shape, and location of any type of liver or abdominal tumor, as well as the blood arteries that surround them. CT scans can also be used to precisely direct a biopsy needle into a potentially cancerous tumor [2]. MRI scans produce comprehensive images of the soft tissues of the body. In addition, they employ radio waves and powerful magnets rather than X-rays. MRI scans can be very useful in the investigation of liver tumors. They can occasionally distinguish between benign and malignant tumors. They can also be used to detect blockages in the blood arteries in and around the liver, as well as to determine whether liver cancer has migrated to other parts of the body [3,4]. Ultrasound is usually used as the initial diagnosis when examining the liver. It generates an image on a computer screen by using sound waves. This test can detect liver tumors, which can then be evaluated for cancer if necessary [5].

Despite the importance of the histological classification of hepatocellular carcinoma, the clinical diagnosis, treatment, prognosis, and grading assessment of liver cancer from radiographs present challenges for medical professionals. Therefore, they must rely heavily on their previous experience, noting that, with the emergence of medical image analysis techniques in recent years and the great scientific progress and reliance on computers in various areas of life, it has become necessary to develop computer-aided diagnosis (CAD) systems to ease the burden on doctors and specialists by automatically classifying liver cancer subtypes. Hence, the importance of this study is to propose a method for detecting liver cancer with the help of artificial intelligence (AI) and deep learning. AI is defined as an ability of a computer to perceive the surrounding environment and make the same decisions as a human intellect on an action to reach a particular goal [6].

Recently, AI has played an important role in different applications [7,8,9,10,11], especially medical applications [12,13,14,15,16]. AI approaches for liver cancer detection are divided into two main approaches, namely the AI-based machine learning approaches [17,18,19,20,21] and AI-based deep learning approaches [22,23,24,25,26,27,28,29,30,31]. The first approach, which relies on machine learning, has several shortcomings, such as obtaining low accuracy using big data, suffering from overfitting or underfitting problems, complex methods, and being time-consuming. Currently, deep learning approaches are widely used in medical applications, which overcome most machine learning approaches, especially when working on big datasets. Furthermore, several recent review studies [32,33,34,35] in this field show that almost all recent works in this field have focused on deep learning. Therefore, this paper focuses on using an AI-based deep learning approach.

Das et al. [22] presented a system based on deep learning approaches for liver cancer detection. They used the watershed transform to separate the liver from other parts of the body and the Gaussian mixture model (GMM) for cancer segmentation. They obtained a classification accuracy of 99.38% after 200 epochs using a deep neural network as a classifier. Ghoniem [23] introduced a bio-inspired approach based on deep learning for the diagnosis of liver cancer. The author used a hybrid segmentation algorithm from several models, such as artificial bee colony optimization and U-Net Network, to extract liver lesions from CT images. Finally, the author used another hybrid algorithm for feature extraction and classification, with an accuracy of 98.5%. Dong et al. [24] presented a method based on deep learning using hybridized full CNN for liver cancer detection and lesion segmentation. For feature extraction, they used several layers as feature extractors and combined these features with several slices. They obtained an overall accuracy of 97.22% for cancer detection. Sureshkumar et al. [25] presented a method using deep learning techniques for detecting a tumor in the liver. One of the deep methods presented in this study is a probabilistic neural network (PNN) for detecting and diagnosing liver tumors. They found that the PNN method improved the overall accuracy with limited features, compared with other machine learning methods. Kaur et al. [26] presented a multi-organ classification technique based on CNN using 3D CT images for liver cancer. They introduced this method to reduce the high computational cost of deep learning. They obtained an accuracy of 99.1% when using data augmentation techniques. Shukla et al. [27] used the cascaded CNN method for liver cancer prediction. First, they performed end-to-end liver segmentation to reduce the error rate during the training process. After that, they applied the presented method to the liver segmentation images. They obtained an accuracy of 94.21 using the presented method. However, most of these studies are not robust and obtain low performance, such as the studies of Kaur et al. [26] and Shukla et al. [27], which need analysis techniques to increase the robustness of the proposed models. Furthermore, when working with small datasets, some of these works obtained low classification results, such as the studies of Das et al. [22], Ghoniem [23], and Kaur et al. [26]. In addition, some of the works are of computational complexity, such as those of Das et al. [22], Dong et al. [19], and Sureshkumar et al. [25].

However, the majority of the current studies that used AI-based deep learning techniques in the field of liver tumor detection focused on the effect of using one pre-trained model and comparing it with other models, thus obtaining classification accuracy. In this study, we overcome these issues by proposing a new and efficient integration method that combines several CNN models for liver tumor detection. The proposed method outperforms most of the previous models using small and big data. Moreover, our method is more robust than previous deep learning methods for liver detection with low computational requirements. Hence, the importance of our study lies in integrating several pre-trained models to detect liver tumors. The novel contributions of this paper are proposing a new integration method that combines several CNN models for liver tumor detection. The proposed method achieved acceptable performance, compared with other deep learning approaches in this field. In addition, we applied the data source to a set of pre-trained deep neural networks and compared them. Moreover, we proposed an algorithm that is able to achieve good results on classification tasks, which is an important component for the development of automated computer-aided systems for liver tumor detection. Finally, we developed an experiment for the proposed method using an abdominal CT scan dataset extracted from the Liver Tumor Segmentation Challenge (LiTS17) [36], and other CT scans from the 3D-IRCADb-01 database [34]. The results demonstrate that our model outperforms most of the current models, especially when working on small datasets.

The rest of the paper is organized as follows: Section 2 illustrates the proposed method adopted in this work. Experimental results and analysis are shown in Section 3. A more comprehensive discussion can be found in Section 4. Finally, Section 5 concludes the paper and provides insights about future work.

## 2. Methodology

**Figure 1:**
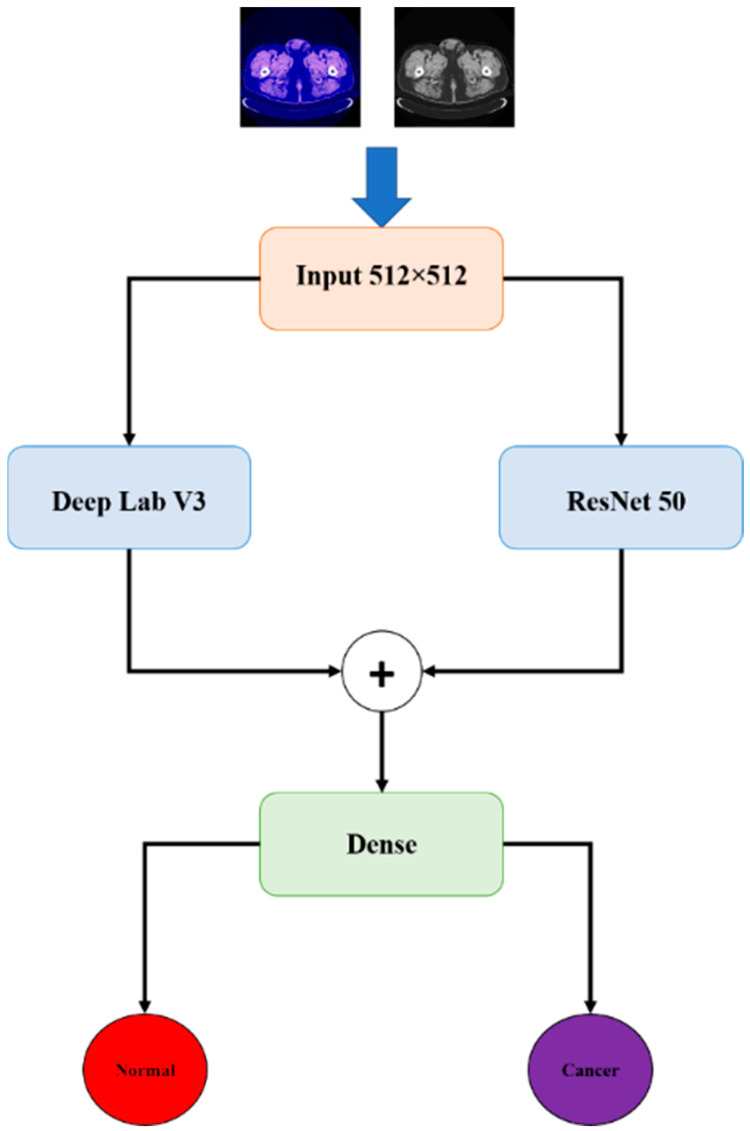
The components of the proposed model.

In this section, we discuss the databases used with our model. In addition, the proposed method is discussed in detail. The general block diagram of the proposed method is shown in Figure 1.

### 2.1. LiTS17 Dataset

In this study, the LiTS17 dataset [36] from the challenge of liver tumor segmentation was employed to evaluate our method. This dataset is a training case for livers and tumor lesions. It is made up of 130 CT scans.

### 2.2. 3D-IRCADb-01 Dataset

This database [34] consists of 20 3D CT scans from 20 different patients, with liver tumors in 75% of cases. The size of each image is 512 × 512 pixels, with different liver average densities between 40 and 135. The labeled images were segmented in a DICOM format. In this study, 10% of the total CT scan images were separated for testing; for this purpose, 26 CT images were randomly selected with the help of Python libraries. Additionally, 10% of the training images were separated for validation; that is, 80% of the training images were preserved, and 20% of them were evaluated on the remaining training image samples after separating the test sample, meaning that we had 80% training images, 10% testing images, and 10% evaluation images.

### 2.3. Data Augmentation

**Figure 2:**
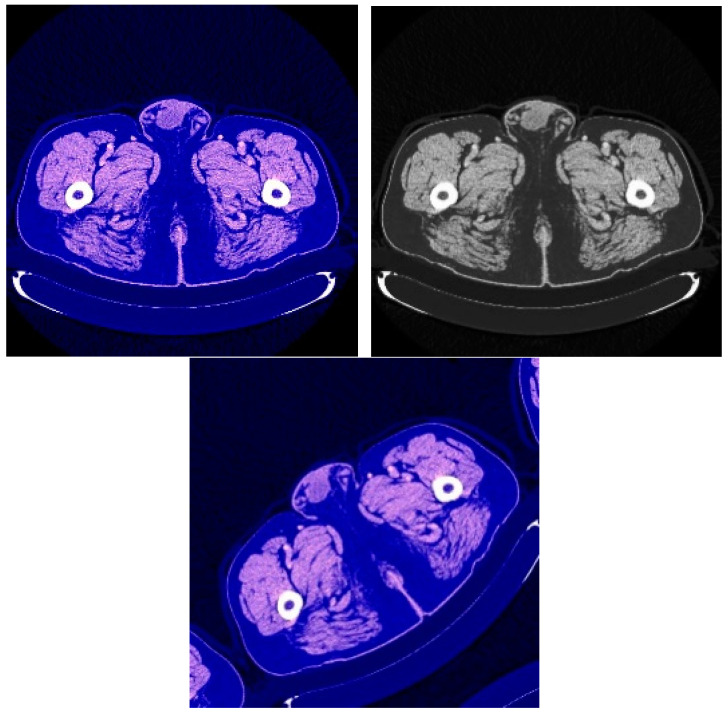
Examples of the input CT images after applying augmentation.

New images were generated in order to enhance the training dataset to allow the proposed network to visualize a greater diversity of tumors by replacing some images through a data augmentation method that works on the following properties: Horizontal Flip, Channel Shuffle, Coarse Dropout, Center Crop, Crop, and Rotate. Figure 2 shows several examples of the images after applying this augmentation technique. In this way, the training and testing samples were enhanced by adding images with new properties derived from the basic set but subject to one of the previously existing processes. Increasing the data in this way allowed for testing the extent to which the resulting models are able to recognize images if they are subject to rotation or enlargement processes. The models save the shapes and give correct results when they are in a certain position or are able to understand the shape even if it is upside down.

### 2.4. Proposed Learning Model

Through a review of the literature, we found that all the studies that rely on deep learning using pre-trained neural networks were based on one model or comparisons between several models. Therefore, in this paper, we present a detailed explanation of our proposed model that integrates several pre-trained models and takes advantage of the different characteristics that each model may have. Classification is the essence of computer vision, and traditional methods describe and discover features, but they are not effective in complex classifications, so they are no longer used. At present, deep learning methods such as CNNs have been adopted, which have made very important contributions in the field of tumor determination when used to classify images. Therefore, in this paper, we tested two combinations of pre-trained models on both datasets and then selected the best combination to be our method. The combinations are as follows:

*First*, we combined DeeplapV3 [37] with ResNet-50 [38]. This combination is used to detect other types of cancer such as breast cancer, etc. [39,40,41], and in other applications, such as those presented in [42]. *Second*, we combined VGG-16 [43] with ResNet-50V2 [44], and U-Net [45] with LSTM [46]. This combination is used in other medical applications, as presented in [47,48,49]. These models were employed as pre-trained models to take advantage of their prior knowledge and the weights found by using them for liver detection.

#### 2.4.1. First Model (DeeplapV3 + ResNet-50)

**Figure 3:**
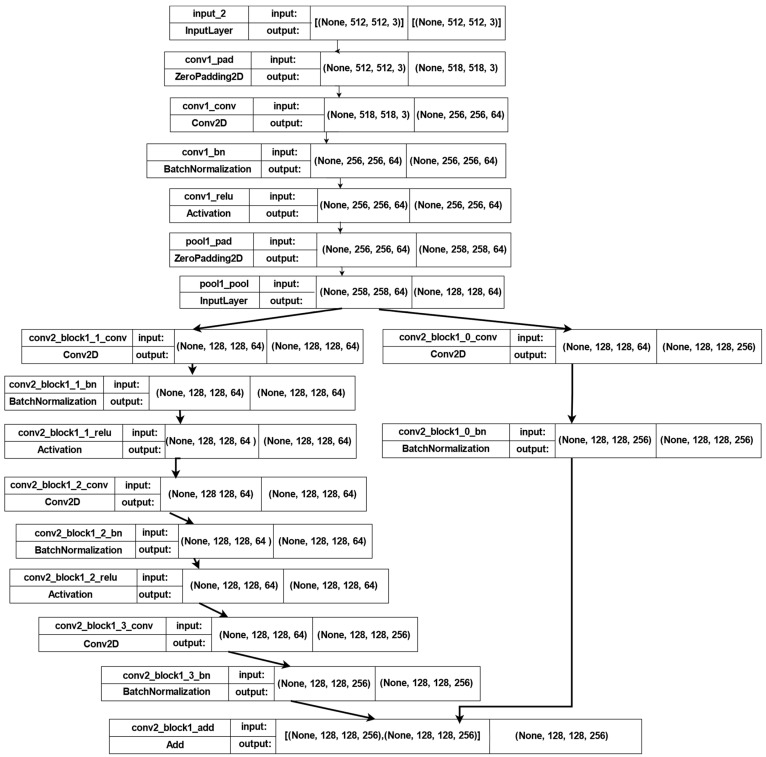
The first part of the architecture of the first model.

In this model, we employed DeeplapV3 [37] and ResNet-50 [38] as pre-trained models on the ImageNet image collection to take advantage of their foreknowledge and the weights found by using them. Figure 3 shows the first part of the model, in which the input size is 512 × 512, with a total parameter value of 17,869,697. This model consists of *nine* blocks. The *first* block contains *one* 2D convolution layer, *one* 2D max-pooling layer, and *one* batch normalization layer with a ReLU activation function. The second block contains 10 2D convolution layers, 3 additional layers, and 10 batch normalization layers, followed by ReLU activation functions. The third block consists of 13 2D convolution layers, 4 additional layers, and 13 batch normalization layers, followed by ReLU activation functions. The fourth block consists of 19 2D convolution layers, 6 addition layers, and 19 batch normalization layers, followed by ReLU activation functions. The next block consists of *one* average pooling layer and *four* 2D convolution layers, followed by *four* batch normalization layers, and *one* concatenation layer followed by *four* activation layers. Block number 6 contains *two* 2D convolution layers, followed by *two* batch normalization layers; *one* concatenation layer, followed by *one* activation layer; and *one* global average 2D pooling layer, followed by *two* dense layers. The remaining blocks each consist of *one* 2D convolution layer, followed by *one* batch normalization layer with an activation function. In addition, there is *one* global average 2D pooling layer, followed by *two* dense layers.

DeepLabv3 was utilized to solve the problem of segmenting objects at different scales. Modules were built that use atrous convolution in cascade or parallel to capture a multi-scaled context by employing multiple atrous rates. The original ResNet study was primarily motivated by the desire to address the degradation problem in a deep network. Adding extra layers to a suitably deep neural network causes accuracy to reach saturation and subsequently decrease. The residual block F(x) can be mathematically represented as follows:

(1) $$y=F\left(x,\left\{W_{i}\right\}\right)+x$$

where y is the output function, x is the input to the residual block, and F(x, {W_i_}) is the residual block.

##### Layers in Block 8

The following layers were added to the model:

- Global average 2D pooling layer: Fully connected for downsampling operation, which was named the concatenate layer;
- Addition layer: Adding the output features of the layers in the same block, which was named the “add layer”;
- The output layer is of the dense layer type, which consists of the neurons of the output rows.

#### 2.4.2. Second Model (VGG-16 + ResNet-50V2 + U-Net + LSTM)

**Figure 4:**
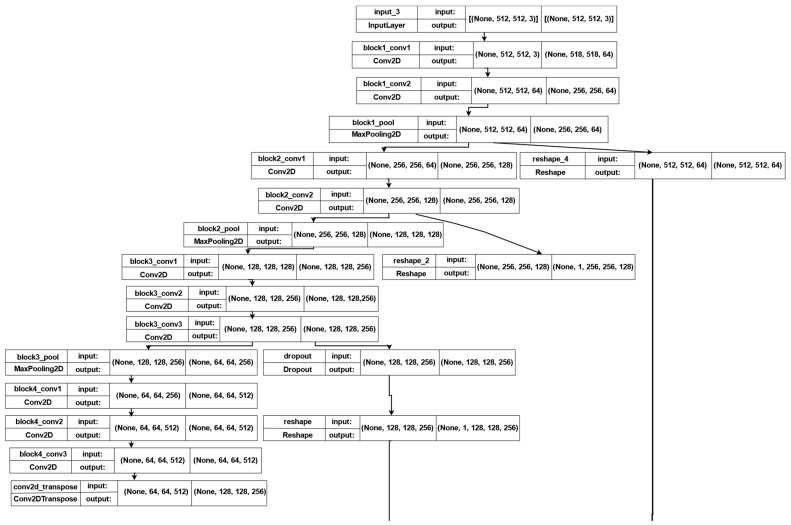
The first part of the architecture of the second model.

In this model, we employed VGG-16 [43], ResNet-50V2 [44], U-Net [45], and LSTM [46] as pre-trained models. Figure 4 shows the first part of the model, in which the input size is 512 × 512, with a total parameter of 11,829,569. This model consists of *seven* blocks. The *first* and *second* blocks contain *two* 2D convolution layers and *one* 2D max-pooling layer for each block. The *third* block contains *three* 2D convolution layers, followed by *one* 2D max-pooling layer. Block number 4 consists of *two* 2D convolution layers and *one* batch normalization layer, followed by *one* dropout layer with a ReLU activation function. In addition, this block has *one* concatenation layer, followed by *two* 2D convolution layers, *two* transpose layers, *one* batch normalization layer, and *three* 2D convolution LSTM layers. The other blocks have the same structure as block 4. The following layers were added to the model:

- Global average 2D pooling layer: Fully connected for downsampling operation, which was named as concatenate layer;
- Dropout layer: Deletes or drops some units from the network to avoid the problem of overfitting by 0.5, which was named the “dropout layer”;
- Transpose layer: Transposes the weights and flips them by 180 degrees, which was named the Conv2DTran layer;
- The 2D convolution LSTM layer: Similar to an LSTM layer, but the input transformations and recurrent transformations are both convolutional, which was named the ConvLSTM2D layer.

VGG-16 is a common image classification method that works well with transfer learning. This algorithm is easy to comprehend and explain, and it produces a solid enough baseline to yield a high classification score. U-net was developed and first used for biomedical image segmentation. Its architecture is roughly equivalent to an encoder network, followed by a decoder network. LSTM can recall long-distance dependency links in a short amount of time, solves the problem of feature loss in conventional recurrent neural networks when longer time steps are encountered, and performs better in longer sequences. A single LSTM cell is made up of numerous systems, including an input gate, a memory cell, a forget gate, an output gate, and a hidden state. The LSTM cell’s mathematical model is as follows:

(2) $$f_{t}=\sigma \left(w_{\mathrm{fx}}\times x_{t}+w_{\mathrm{fh}}\times h_{t-1}+b_{f}\right)$$

(3) $$i_{t}=\sigma \left(w_{\mathrm{ix}}\times x_{t}+w_{\mathrm{ih}}\times h_{t-1}+b_{i}\right)$$

(4) $$\tilde{c_{t}}=\tanh\left(w_{\mathrm{cx}}\times x_{t}+w_{\mathrm{ch}}\times h_{t-1}+b_{c}\right)$$

(5) $$c_{t}=f_{t}\times c_{t-1}+i_{t}\times \tilde{c_{t}}$$

(6) $$o_{t}=\sigma \left(w_{\mathrm{ox}}\times x_{t}+w_{\mathrm{oh}}\times h_{t-1}+b_{o}\right)\;$$

(7) $$h_{t}=o_{t}\times \tanh\left(c_{t}\right)$$

where b_f_, b_i_, b_c_, and b_o_ are the bias values of the forget gate, the memory gate, the cell state, and the input gate, respectively. $\tilde{c_{t}}$ is the temporary cell state, c_t__−1_ is the cell state at the previous moment, and h_t_ is the hidden layer state. σ is the activation function of the gating structure, which is applied to memory and forgetting of the information, and the tanh activation function is used to determine the temporary cell state $\tilde{c_{t}}$ and the hidden state h_t_, which can be replaced for data processing.

## 3. Experimental Results and Analysis

The models were executed in Python based on the TensorFlow with the Keras library, which offers high-level packaging for different layers. We evaluated both models on the two datasets, and then the model that achieved the best performance was confirmed to be our model. After that, we compared the selected model with other previous models in this field.

### 3.1. Performance Metrics

To validate the proposed model, accuracy (Acc), precision (P), recall (R), and an even Dice coefficient (dice_cof) were selected as evaluation metrics, which are defined as follows:

(8) $$\mathrm{Acc}=\frac{\mathrm{TP}+\mathrm{TN}}{\mathrm{TP}+\mathrm{TN}+\mathrm{FP}+\mathrm{FN}}$$

(9) $$P=\frac{\mathrm{TP}}{\mathrm{TP}+\mathrm{FP}}$$

(10) $$R=\frac{\mathrm{TP}}{\mathrm{TP}+\mathrm{FN}}$$

(11) $${\mathrm{dice}}_{\mathrm{cof}}=\frac{2\mathrm{TP}}{\left(2\mathrm{TP}+\mathrm{FP}+\mathrm{FN}\right)}$$

where TP = true positives, FP = false positives, FN = false negatives, and TN = true negatives.

### 3.2. Results of the LiTS17 Dataset

In this section, we discuss and analyze the results of the proposed two models using the LiTS17 dataset.

- *The results of the first proposed model*

**Figure 5:**
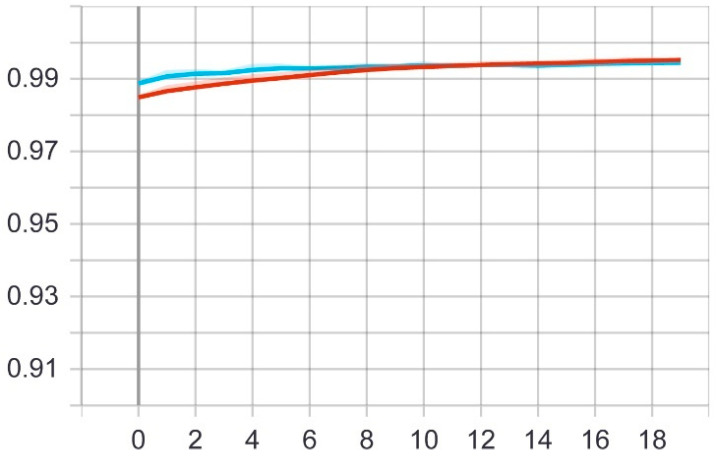
Accuracy (red line) and validation (blue line) convergence curves of the first model over 20 epochs.

At this stage, the first proposed model was tested on the test set that was previously divided randomly from the dataset. The model was trained on 20 epochs according to the previous set of data and divisions, and as shown in Figure 5, the drawing of the accuracy curve and the validation accuracy curve converges, providing evidence that the training stabilized after 10 epochs, and there was an increase in accuracy.

**Figure 6:**
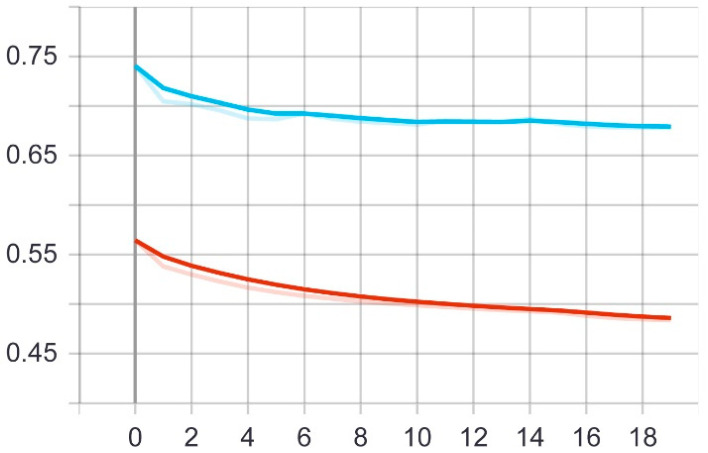
Accuracy (red line) and validation (blue line) loss curves of the first model over 20 epochs.

Through these functions, the model was trained on the training sample and then checked its results against the aforementioned test sample, which improved the values of the hidden coefficients for the last layer in the model in order to match the images to be trained in each phase. This process was repeated for all training epochs, and in epochs 14 and above, it was noticed that this accuracy did not improve more than a certain threshold, meaning that every time the model was trained on a set of images from the training sample, it checked images in the validation sample and obtained a certain accuracy. In this case, the accuracy reached 0.995, meaning that the accuracy of the model during the training and validation phases was 99.5%. The loss curve is also shown in Figure 6.

**Figure 7:**
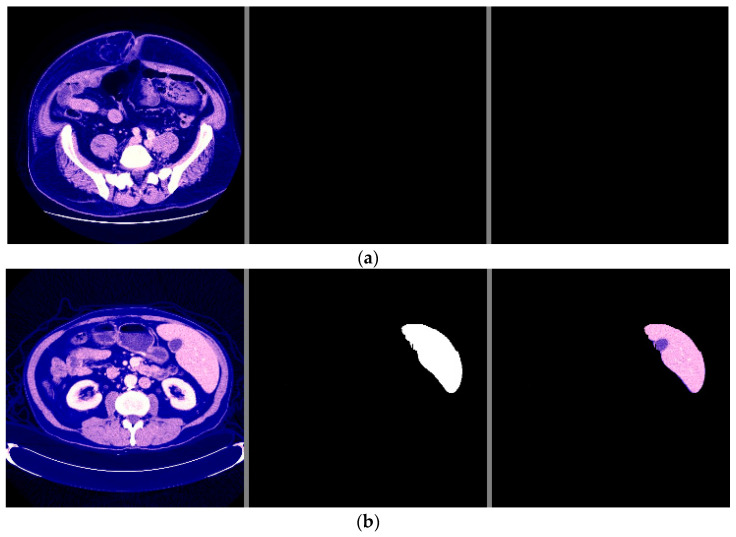
The visual results of the first model for cancer detection on LiTS dataset: (**a**) normal case (no tumor detection); (**b**) abnormal case (tumor detection).

The visual examples of the results of the model for detecting liver cancer from the dataset are shown in Figure 7.

The overall performance based on the metrics is shown in Table 1.

- *The results of the second proposed model*

**Figure 8:**
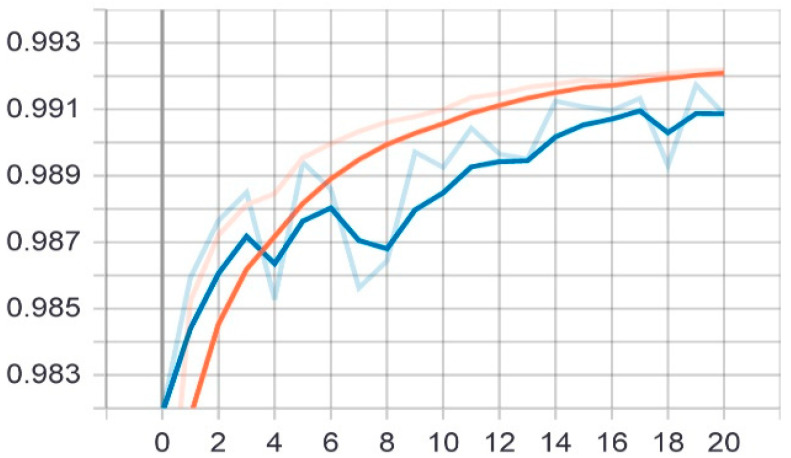
Accuracy (red line) and validation (blue line) convergence curves of the second model over 20 epochs.

The second proposed model was tested on the same test set that was applied to the first model. The model was trained on 20 epochs according to the previous set of data and divisions, and as shown in Figure 8, the drawing of the accuracy curve and the validation accuracy curve converged, providing evidence that the training stabilized after 16 epochs, and there was an increase in accuracy. Through these functions, the model was trained on the training sample and then checked its results against the aforementioned test sample, which improved the values of the hidden coefficients for the last layer in the model in order to match the images to be trained in each phase.

**Figure 9:**
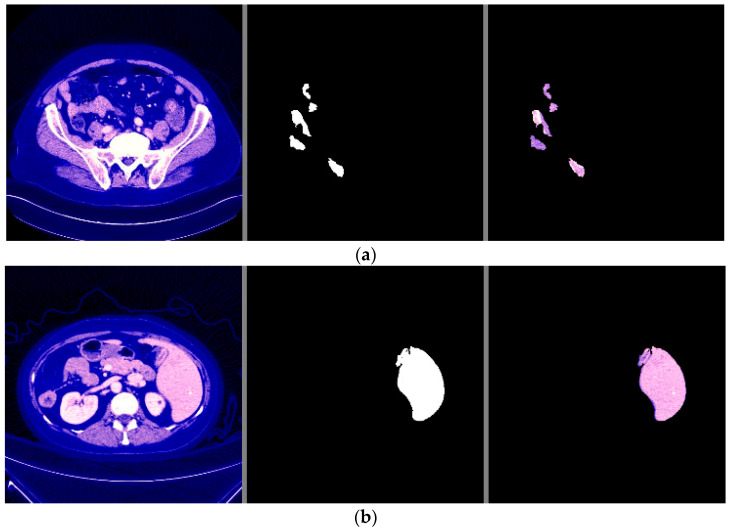
The visual results of the second model for cancer detection on LiTS dataset: (**a**) normal case (no tumor detection); (**b**) abnormal case (tumor detection).

This process was repeated for all training phases, and in phases 18 and above, it was noticed that this accuracy did not improve more than a certain threshold, meaning that every time the model was trained on a set of images from the training sample, it checked images in the validation sample and obtained a certain accuracy. In this case, the accuracy reached 0.991, meaning that the accuracy of the model during the training and validation phases was 99.1%. The visual examples of the results of the model for detecting liver cancer from the dataset are shown in Figure 9.

**Figure 10:**
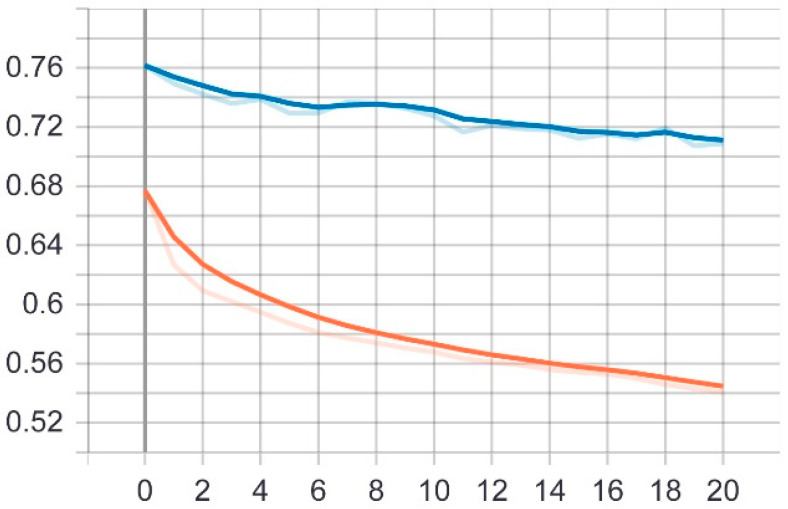
Accuracy (red line) and validation (blue line) loss curves of the second model over 20 epochs.

The loss curve of the second model is also shown in Figure 10.

The overall performance based on the metrics is shown in Table 2.

### 3.3. Results of the 3D-IRCADb-01 Dataset

The same experiments were performed on the second dataset (3D-IRCADb-01) for the two models.

- *The results of the first proposed model*

**Figure 11:**
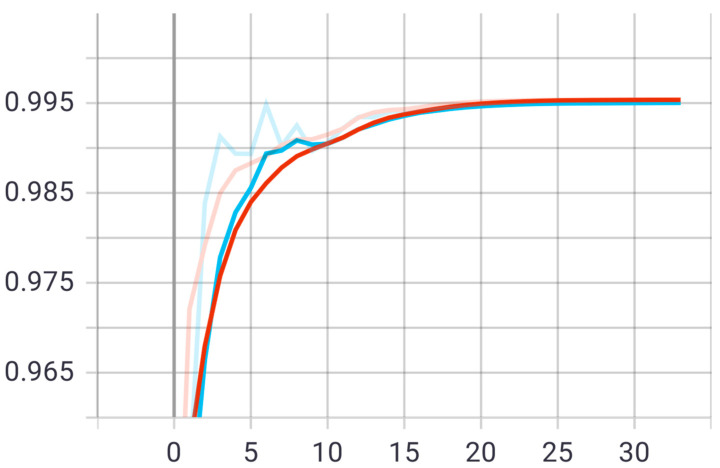
Accuracy (red line) and validation (blue line) convergence curves of the first model over 30 epochs.

Within this stage, the first proposed model was tested on the test set that was previously divided randomly from the dataset. The model was trained on 30 epochs according to the previous set of data and divisions, and as shown in Figure 11, the drawing of the accuracy curve and the validation accuracy curve converged, providing evidence that the training stabilized after 10 epochs, and there was an increase in accuracy.

**Figure 12:**
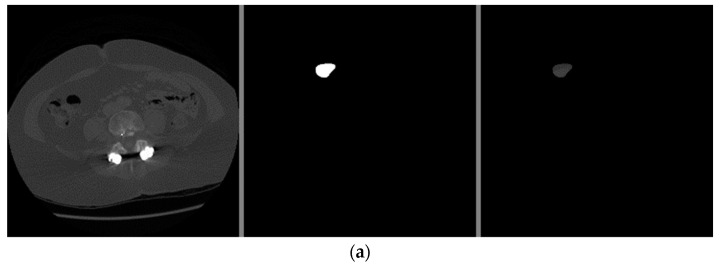

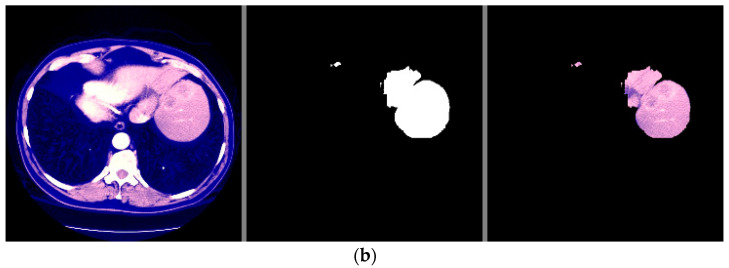
The visual results of the first model for cancer detection on 3D-IRCADb-01 test set: (**a**) normal case (no tumor detection); (**b**) abnormal case (tumor detection).

Through these functions, the model was trained on the training sample and then checked its results through the aforementioned test sample, which improved the values of the hidden coefficients for the last layer in the model in order to match the images to be trained in each phase. This process was repeated for all training phases, and in phases 15 and above, it was noticed that this accuracy did not improve more than a certain threshold, meaning that every time the model was trained on a set of images from the training sample, it checked images in the validation sample and obtained a certain accuracy. In this case, the accuracy reached 0.995, meaning that the accuracy of the model during the training and validation phases was 99.5%. The visual examples of the results of the model for detecting liver cancer from the dataset are shown in Figure 12.

**Figure 13:**
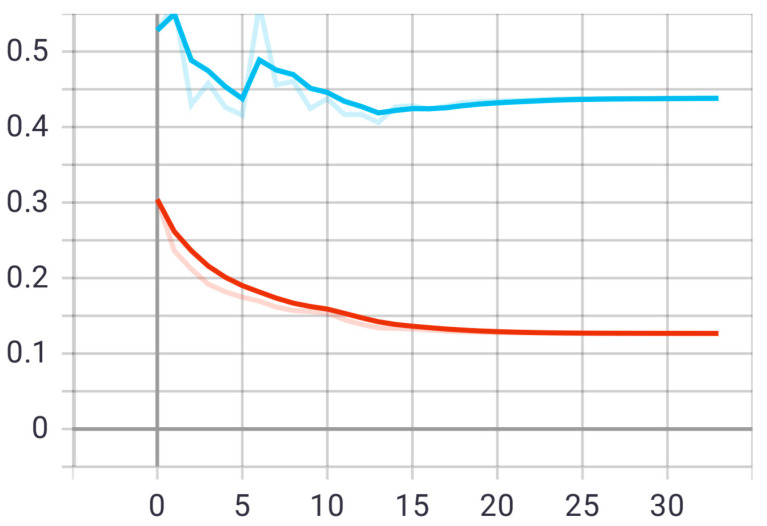
Accuracy (red line) and validation (blue line) loss curves of the first model over 30 epochs.

The loss curve of the second model is also shown in Figure 13.

The overall performance based on the metrics is shown in Table 3.

- *The results of the second proposed model*

**Figure 14:**
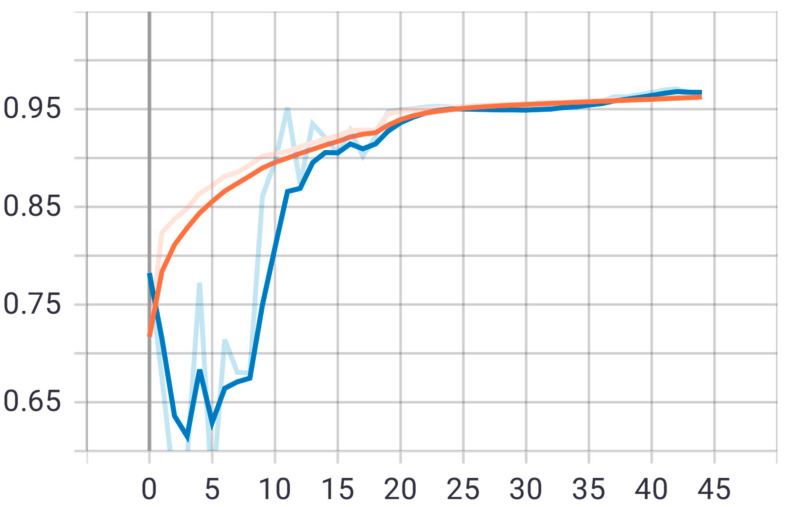
Accuracy (red line) and validation (blue line) converge curves of the second model over 45 epochs.

**Figure 15:**
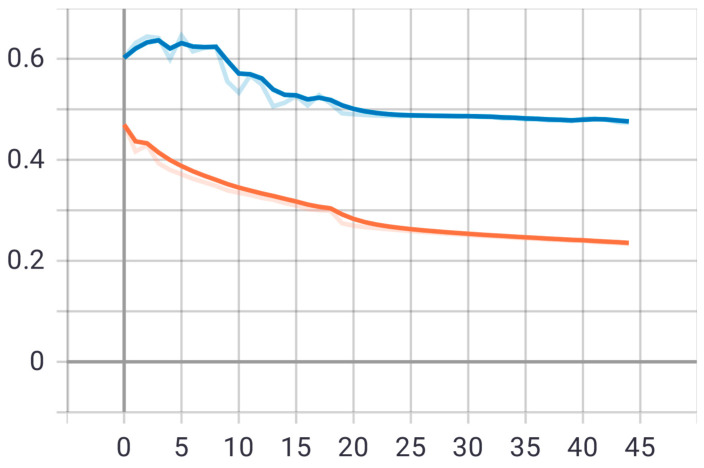
Accuracy (red line) and validation (blue line) loss curves of the second model over 45 epochs.

The second proposed model was tested on the same test set that was applied to the first model. The model was trained on 45 epochs according to the previous set of data and divisions, and as shown in Figure 14, the drawing of the accuracy curve and the validation accuracy curve converged, providing evidence that the training stabilized after 20 epochs, and there was an increase in accuracy. Through these functions, the model was trained on the training sample and then checked its results through the aforementioned test sample and improved the values of the hidden coefficients for the last layer in the model in order to match the images to be trained in each phase. This process was repeated for all training epochs, and in epochs 25 and above, it was noticed that this accuracy did not improve more than a certain threshold, meaning that every time the model was trained on a set of images from the training sample, it checked images in the validation sample and obtained a certain accuracy. In this case, the accuracy reached 0.984, meaning that the accuracy of the model during the training and validation phases was 98.4%. The loss curve of the second model is also shown in Figure 15.

**Figure 16:**
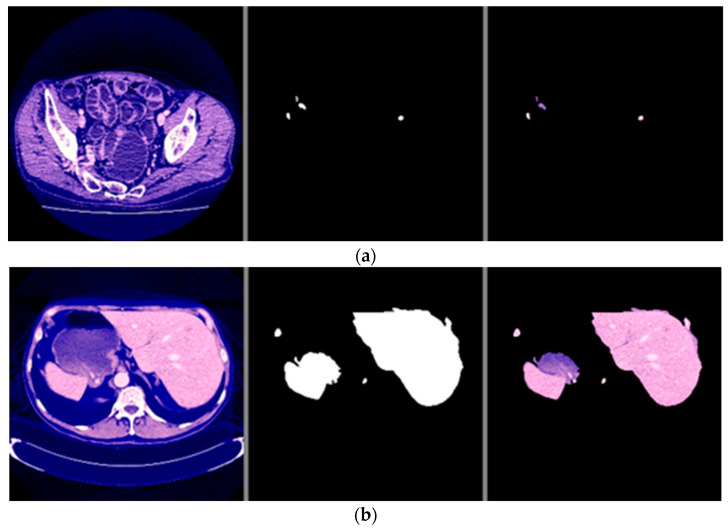
The visual results of the second model for cancer detection on 3D-IRCADb-01 dataset: (**a**) normal case (no tumor detection); (**b**) abnormal case (tumor detection).

The visual examples of the results of the model for detecting liver cancer from the dataset are shown in Figure 16.

The overall performance based on the metrics is shown in Table 4.

## 4. Discussion

From the previous results mentioned in Section 3, it was observed that the first proposed method (the combination of DeeplapV3 and ResNet 50) achieved better accuracies than the second method (the combination of VGG-16, ResNet 50 V2, U-Net and LSTM) using both datasets. Therefore, we confirmed the first method to be our method. We also observed from the results that the first method obtained a high accuracy of 99.5% on small data (the case of the second dataset), which makes it more robust and efficient than other previous deep learning methods. In addition, when using the first dataset, as shown in Figure 7, it was observed that the first model could accurately detect cancer from the normal image. However, we can see from Figure 9 that the second model made some noise when detecting cancer from a normal image (as shown in Figure 9a). In terms of the second dataset (small data), we can observe from Figure 12 and Figure 16 that, when detecting cancer from the image, the two models detected cancer with some noise, but the first model was still better than the second model, with less noise. We employed the ReLU activation function in our models because it does not become active in all neurons at the same time. In addition, ReLU gives the highest accuracy, compared with other nonlinear activation functions, such as the Tanh activation function. Table 5 shows a performance comparison when using ReLU as an activation function and other nonlinear activation functions on the 3D-IRCADb-01 dataset.

As evident in Table 5, it was observed that using ReLU as an activation function resulted in better performance than other nonlinear activation functions such as leaky ReLU and Tanh activation functions. Therefore, we selected ReLU as an activation function in our models in all cases.

The liver cancer detection system, with the help of AI, is an assistant system for doctors to determine whether a person is infected, which greatly helps by reducing the occurrence of errors in medical diagnosis and helps in saving time and effort for the doctor, as it gives faster and more accurate results. Thus, it contributes to reducing the number of deaths from liver cancer. Therefore, several studies presented different models for liver cancer detection [22,23,24,25,26,27]. After reviewing a set of research studies aimed at transferring learning from previously trained models on the same datasets used in this study, a set of previously trained models were studied, tested, and compared with the proposed method in terms of accuracy and other metrics, as shown in Table 6.

Despite the presence of many contributions in this field, all of them focused on the use of a single neural network to detect cancer, and there is still no attempt to combine a number of models with each other to take advantage of the different properties of each model. In addition, most of the previous works obtained low results using small data, unlike our method, which achieved very good results with small data.

This research had several restrictions. In order to fit the Nvidia 1080 Ti GPU (8 GB Detected Memory), CT scans were shrunk. Future research can train models utilizing more significant image sizes or keeping the original image resolution without needing downscaling as additional GPU memory becomes available. Consequently, the segmented tumor area is shown in more detail, and performance is probably improved by maintaining the full resolution of contemporary digital CT scan images. The LITS dataset and 3D-IRCADb-01 dataset did not contain all cancer tumors, and we used radiologists’ assessments to assign labels to the images in the datasets. Future research on cases of interval liver cancer that radiologists missed and MRI datasets would be interesting to train algorithms to detect more subtle cancer tumors that might not be visually obvious.

Lastly, the LITS and 3D-IRCADb-01 datasets did not contain nationally representative samples. Thus, the performance metrics in these datasets cannot be directly compared to estimates of the sensitivity and specificity of radiologists at the national level.

This research also had limitations in terms of the massive number of weight parameters, resulting in a big model size and inference time.

## 5. Conclusions

In this paper, we proposed a liver cancer detection system from CT images using deep learning techniques and pre-trained models. In addition, we provided a reference study for the most recent work dealing with these deep-learning-based systems. The architecture of the proposed models is characterized by creating a new architecture capable of leveraging the power of several pre-trained models and transferring learning from them. We selected the best model, which is presented as capable of learning using two pre-trained CNNs and integrating the outputs of the last layer of each of these networks. Then, this model was adjusted to be able to recognize images of liver tumors. The proposed model achieved an accuracy of 99.5% on both datasets, which is better than the results of other models used in this field. The proposed network model can be used in the liver cancer diagnosis and treatment system, which is expected to help clinicians make better surgical plans for liver cancer patients. We plan to improve our model as a follow-up study in the future in order to obtain better performance and, thus, provide a more accurate classification. We also aim to test the model on other types of cancer and measure the accuracy of this model. Finally, we aim to evaluate other activation functions, similar to those in [50], and observe the results of the proposed approach.

## Figures and Tables

**Table 1:** The overall performance of the first model on LiTS dataset.

Acc	P	R	Dice_cof
0.995	0.864	0.979	0.516

**Table 2:** The overall performance of the first model on LiTS dataset.

Acc	P	R	Dice_cof
0.991	0.778	0.846	0.291

**Table 3:** The overall performance of the first model on 3D-IRCADb-01 dataset.

Acc	P	R	Dice_cof
0.995	0.514	0.986	0.561

**Table 4:** The overall performance of the second model on 3D-IRCADb-01 dataset.

Acc	P	R	Dice_cof
0.984	0.735	0.802	0.405

**Table 5:** Comparison of our method using ReLU activation function and other nonlinear activation functions.

Activation Function	Performance (%)
ReLU	Acc = 99.50 Dice_cof = 51.64 P = 86.41 R = 97.92
Leaky ReLU	Acc = 98.84 Dice_cof = 24.45 P = 75.52 R = 21.50
Tanh	Acc = 98.43 Dice_cof = 26.19 P = 73.78 R = 22.45

**Table 6:** Comparison of the performance of our method and those of other previous methods (our results in **bold**).

Author	Methodology	No. Images	Accuracy
Das et al. [22]	WGDL+ GMM+ DNN	225 CT images	99.39%
Ghoniem [23]	LeNet-5/ABC	131 CT images	98.50%
Dong et al. [24]	HFCNN	N/A	97.22%
Kaur et al. [26]	CNN	63503 CT images	99.10%
Shukla et al. [27]	Cascaded CNN	1421 CT images	94.21%
**Proposed**	**DeeplapV3 + ResNet-50**	**130 CT images** **LITS dataset** **and** **26CT images 3Dicdb dataset**	**99.50%**

## Data Availability

No new data were created or analyzed in this study. Datasets are available at 1. https://competitions.codalab.org/competitions/17094, 2. https://www.ircad.fr/research/data-sets/liver-segmentation-3d-ircadb-01/.
